# Hydrogel Contact Lenses Embedded with Amine-Functionalized Large-Pore Mesoporous Silica Nanoparticles with Extended Hyaluronic Acid Release

**DOI:** 10.3390/nano13172441

**Published:** 2023-08-28

**Authors:** Chun-Feng Lai, Fu-Jia Shiau

**Affiliations:** Department of Photonics, Feng Chia University, Taichung 407, Taiwan

**Keywords:** contact lenses, mesoporous particles, silica, nanoparticles, hyaluronic acid

## Abstract

Contact lenses (CLs) have emerged as an effective method for delivering ophthalmic drugs. In this research, we designed hydrogel CLs capable of extended release, utilizing large-pore mesoporous silica nanoparticles (LPMSNs) to deliver hyaluronic acid (HA) for treating dry eye syndrome. LPMSNs were functionalized with amine groups (LPMSN–amine) to enhance HA loading and release capacity. In vitro release studies demonstrated that LPMSN–amine CLs exhibited superior slower HA release than LPMSN–siloxane and standard CLs. Within 120 h, the cumulative amount of HA released from LPMSN–amine CLs reached approximately 275.58 µg, marking a 12.6-fold improvement compared to standard CLs, when loaded from 0.1 wt% HA solutions. Furthermore, LPMSN–amine CLs effectively maintained moisture, mitigating ocular surface dehydration, making them a promising solution for dry eye management. This study successfully developed LPMSN–amine CLs for extended HA release, identifying the optimal functional groups and loading conditions to achieve sustained release.

## 1. Introduction

Dry eye syndrome, also referred to as keratoconjunctivitis sicca (KCS), is a condition characterized by insufficient tears to lubricate the conjunctiva and cornea adequately, resulting in discomfort. The Tear Film and Ocular Surface Society Dry Eye Workshop II report states that approximately over three million people worldwide suffer from KCS. Common symptoms of KCS include dryness, redness, a sensation of burning or stinging, itching, blurred vision, sensitivity to light, eye fatigue, and excessive tearing. Individuals with KCS often rely on eye drops with hyaluronic acid (HA) to improve lubrication and provide relief or apply artificial tears to moisturize their eyes. However, conventional eye drops have certain limitations, such as low bioavailability, short duration of action, need for frequent application, and inconvenience for users, which often lead to low patient compliance [1,2,3,4]. Addressing these issues and improving bioavailability requires a high frequency of drug administration; however, this approach poses the risk of side effects due to potential drug overdose and toxicity [5].

HA is a naturally occurring linear polysaccharide found in the human body. It is known for its unique ability to bind and retain water molecules. Its biocompatibility, biodegradability, and non-immunogenicity have rendered it a versatile component in various biomedical applications, such as tissue engineering, drug delivery, and cosmetic surgery [6,7]. HA’s remarkable hydrophilicity allows its molecules to carry a large amount of water efficiently, promoting water retention. Each HA molecule can carry 500–1000 times its weight in water, making it highly effective in achieving a moisturizing effect to relieve KCS symptoms. Despite these advantages, some products containing HA tend to lose their moisturizing properties over time. Therefore, innovative and breakthrough methods that enable slow and sustained HA release, allowing HA to exert its moisturizing effects fully, are needed. We developed a novel solution in the form of extended-release HA incorporated into hydrogel contact lenses (CLs) embedded with large-pore mesoporous silica nanoparticles (LPMSNs) to address the above challenges. This innovative medical device aims to improve patient compliance and enhance clinical outcomes in the treatment of KCS.

Many works have studied the utilization of CLs for controlled drug delivery with nanoparticles, micelles, microemulsions, and vitamin E [8,9,10,11,12,13,14,15]. In recent years, mesoporous silica nanoparticles (MSNs) have emerged as a highly regarded option owing to their exceptional chemical stability, tunable particle size, and high biocompatibility [16]. Numerous studies have highlighted the remarkable properties of HA, including biodegradability, biocompatibility, and non-immunogenicity, making it an ideal candidate for modifying MSNs [17,18]. Among the different types of MSNs, LPMSNs stand out as particularly promising for controlling drug delivery systems due to their expansive surface area, large pore size, and easy modification [19,20]. Despite these advantages, the combination of LPMSNs with HA to develop long-lasting moisturizing CLs has not been reported in international journals to date. This situation presents an exciting opportunity for our research to explore a novel approach that leverages the benefits of LPMSNs and HA to create advanced CLs with sustained moisturizing properties.

HA typically exists with molecular sizes ranging from 3 kDa to 50 kDa. In this study, we used 3 kDa HA for experimentation. HA with this specific molecular weight has been widely employed in medical research to induce immune inflammatory reactions and angiogenesis and promote wound healing, repair, and tissue reconstruction [21,22]. Additionally, the package storage solutions of commercial CLs incorporate this molecular weight of HA. Unfortunately, the commercial CLs can hardly absorb the HA in packaged storage solutions. This situation piqued our interest in exploring the application of 3 kDa HA in the development of long-lasting moisturizing CLs. However, the pores of conventional MSNs are known to be too small to carry high-molecular-weight HA effectively [16,17]. In this study, we used LPMSNs with pores larger than 30 nm to address this limitation. Our objective is to assess their potential to carry and release HA while developing HA-laden ring-embedded hydrogel CLs for sustained HA delivery without compromising the optical and physical properties of CLs that offer prolonged moisturization.

## 2. Materials and Methods

### 2.1. Experimental Materials

Sodium hyaluronate (HA, eye-drop grade, MW = 3 kDa) was obtained from SeedChem Company Pty., Ltd. (Melbourne, VIC, Australia). 2-Hydroxyethyl methacrylate (HEMA), ethylene glycol dimethyl-acrylate (EGDMA), 2-hydroxy-2-methylpropiophenone (HMPP), reactive Blue 49 (dye), and 2-[3-(2*H*-Benzotriazol-2-yl)-4-hydroxyphenyl]ethyl methacrylate (UV blocker) were provided by Yung Sheng Optical Co., Ltd. (Taichung, Taiwan, also known as HYDRON Co.). Absolute ethanol (EtOH, 99.99%) was obtained from JT Baker Co., Ltd. (Paris, KY, USA). (3-Aminopropyl)trimethoxy-silane (APTES), phosphate-buffered saline (PBS), hydrochloric acid (HCl), sodium chloride (NaCl), sodium bicarbonate (NaHCO_3_), potassium chloride (KCl), and calcium chloride dehydrate (CaCl_2_·2H_2_O) were purchased from Sigma-Aldrich. LPMSNs containing siloxane functional groups were procured from Shengsen Nano Tech. Co., Ltd. (Taichung, Taiwan). These LPMSNs have an average pore size exceeding 30 nm.

### 2.2. Synthesis of Amine-Functionalized LPMSNs

The synthesis of LPMSNs with amine functionalization via APTES linkage followed the methodology described in the literature [17]. Preparation is outlined as follows: first, 0.2 g of LPMSN–siloxane was dissolved in 20 mL of EtOH and dispersed by using ultrasonication for 30 min. Subsequently, 0.18 mL of APTES was added to the solution, and the mixture was refluxed at 80 °C for 24 h. The resulting product was then subjected to centrifugation and washed three times with EtOH to remove any impurities. Finally, the product was dried in a vacuum oven to form LPMSN–amine powder, which was used in this study.

### 2.3. Hydrogel CLs Embedded with LPMSNs

Hydrogel CLs embedded with LPMSNs were prepared in accordance with our previous works [20,23] through the incorporation of LPMSNs functionalized with different groups. In this study, we developed two types of functionalized LPMSN-embedded CLs: LPMSNs with siloxane groups (LPMSN–siloxane CLs) and LPMSNs with amine groups (LPMSN–amine CLs). The process commenced with the creation of the LPMSN ring template, achieved through the self-assembly of LPMSN colloids via solvent evaporation. Subsequently, a 50 μL volume of the hydrogel mixture was introduced into a PP mold containing the LPMSN ring template for subsequent cross-linking. This hydrogel mixture consisted of HEMA (95 wt%), EGDMA (2 wt%), HMPP (1 wt%), dye (1 wt%), and a UV blocker (1 wt%). UV light exposure for 10 min was employed to photopolymerize the mold, solidifying the CLs. The resulting LPMSN-laden CLs were gently removed from the top mold. Subsequently, these CLs underwent a hydration process in a 0.9% NaCl solution for 2 h, followed by rinsing with deionized water. Finally, they were preserved in glass vials containing a PBS solution to ensure their structural integrity and stability. Standard CLs were prepared as reference samples using the same fabrication conditions, except for the omission of LPMSN incorporation. These standard CLs served as a comparison in our experiments, allowing us to assess the effect and advantages of LPMSN functionalization in the development of extended-release hydrogel CLs for improved ophthalmic drug delivery.

### 2.4. Measurement of HA Load and Release

**Scheme 1:**
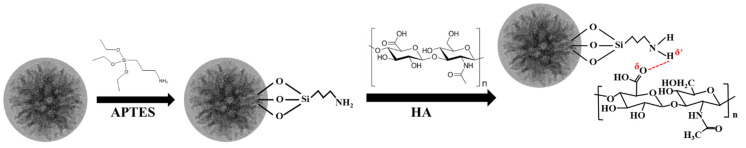
Hydrogen bond routes of the LPMSN–amine loaded HA.

The detailed process for the HA modification of LPMSNs via amine group functionalization is shown in Scheme 1. In this study, we ensured uniform conditions for HA loading and release measurements across all CLs to ensure comparability. These measurements were conducted within a controlled environment in a class 10k clean room, maintaining a constant temperature and relative humidity of 22 °C/50%. First, 0.1 wt% HA/PBS solutions were prepared, and their pH levels were adjusted to 5.5 and 6.5 by using HCl. HA loading involved immersing the CLs in the HA/PBS solutions at pH 5.5 and 6.5 for 24 h. For each measurement of HA release, we utilized samples of LPMSN–siloxane, LPMSN–amine, and standard CLs with six replicates at 22 °C ± 2 °C (*N* = 6). Magnetic stirring was not applied throughout the entire process to replicate real storage conditions. Following sterilization, all HA-loaded CLs were promptly employed for in vitro release investigations. In these experiments, the HA-loaded CLs were placed inside a quartz cuvette containing 3 mL of artificial lacrimal fluid (ALF). It is worth noting that we did not employ any stirring or solvent exchange during this setup, although the quartz cuvette was moved at a velocity of 12 mm/s with a translation stroke of 60 mm [20]. The ALF solution was carefully prepared using a combination of NaCl, NaHCO_3_, KCl, and CaCl_2_·2H_2_O, and its pH was adjusted to 7.4 using HCl [24]. To monitor cumulative drug release, we periodically measured the absorbance of HA/ALF concentration, which was consistently maintained at 3 mL. These measurements were conducted using a UV-Visible (UV-Vis) spectrophotometer to determine the concentration of the released drug, relying on a standard absorbance curve established for HA with a *λ*_max_ at 208 nm. The cumulative amount of released HA was calculated by referencing a calibration curve created from various HA concentrations in ALF. Finally, we analyzed the release profiles by generating graphs illustrating cumulative drug release over time. This meticulous in vitro release study allowed us to evaluate the sustained and controlled release of HA from hydrogel CLs, providing crucial insights into their potential effectiveness for long-term ophthalmic drug delivery.

### 2.5. Release Kinetics Mechanism for LPMSN-Laden CLs

The loading of active molecules onto nanocarrier materials can occur through either physical adsorption or chemical bonding. In this study, we investigated the release kinetics of HA from LPMSNs using the pseudo-first-order and Korsmeyer–Peppas (K–P) models, as described in the literature [25,26,27]. These kinetic models are commonly employed to characterize drug release behavior in nanocarrier systems. Our objective is to predict the in vivo biological performance by utilizing in vitro HA release data. This approach is a rational strategy in the development of controlled release formulations, providing valuable insights into the sustained release of HA from hydrogel CLs and aiding in the optimization of their design for efficient and targeted ophthalmic drug delivery.

The pseudo-first-order kinetic model is defined as Equation (1):

(1) $$Q_{t}\left(t\right)=Q_{e}\left(1-e^{-k_{1}t}\right),$$

where *Q_t_* is the cumulative release percentage of HA at time *t*, *Q_e_* is the release percentage of HA at equilibrium, and *k*_1_ is the release rate constant.

The K-P model is defined as Equation (2):

(2) $$Q_{t}\left(t\right)/Q_{\infty }=k_{kp}\times t^{n},$$

where *Q_t_* is the cumulative release percentage of HA at time *t*, *k_kp_* is the release rate constant, and *n* is a characteristic parameter that represents the HA release mechanism. Values of *n* equal to or less than 0.45 indicate a Fickian diffusion mechanism, those in the range of 0.45 to 0.89 indicate an anomalous drug release mechanism, and those equal to or less than 0.89 indicate a characteristic of matrix erosion.

### 2.6. Characterizations

The functionalized LPMSNs were characterized through various techniques. Their chemical composition was analyzed by using a Fourier-transform infrared spectrometer (FTIR, PerkinElmer Frontier, Shelton, CT, USA). The morphology of the LPMSNs was assessed through the utilization of field-emission transmission electron microscopy equipped with a dark-field function (FETEM, JEM-2100F, JEOL) and field-emission scanning electron microscopy (FESEM, S-4800, Hitachi, Tokyo, Japan). The zeta potentials of the different functionalized LPMSNs were measured by applying a dynamic laser light scattering instrument (DLS, Malvern Instruments Ltd., Newland, UK). Nitrogen adsorption–desorption isotherms were obtained with an adsorption analyzer (ASAP2020, USA). The surface area, pore size, and pore volume of the samples were calculated by employing the Brunauer–Emmett–Teller (BET) and Barrett–Joyner–Halenda (BJH) methods. A UV–Vis spectrophotometer (UV-1900i, Shimadzu Corp., Kyoto, Japan) was employed to measure the HA release rates of CLs. Additionally, water contact angle measurements were conducted by using a contact angle goniometer (FTA-1000B, First Ten angstroms).

## 3. Results and Discussion

### 3.1. Characterization of Functionalized LPMSNs

The original LPMSNs used in this study possessed an internal cross-linked structure of –Si–O–Si– and surface functional groups of siloxane (Si–OH) and were collectively known as LPMSN–siloxane (Scheme 1). The cross-linked structure exhibited almost no chemical activity, whereas the siloxane structure presented relatively low chemical activity and can only undergo reactions with structures containing silicon hydroxyl groups. Therefore, conjugation and adsorption using unmodified LPMSNs directly may result in poor adsorption and controlled release properties due to inadequate interaction forces between the host and guest molecules. We introduced amine functional groups into the LPMSNs to address this limitation and enhance loading, as well as slow-release properties. The resulting product is referred to as LPMSN–amine. The above modification involved altering the surface Si–OH functional groups of the LPMSNs by introducing amine moieties. The addition of amine functional groups improved the interaction forces between the LPMSNs and desired HA molecules, leading to improved loading capacity and controlled release characteristics. We aim to obtain an advanced HA delivery system with enhanced performance offering sustained and targeted HA release for improved therapeutic outcomes by incorporating LPMSN–amine into our hydrogel CLs.

**Figure 1:**
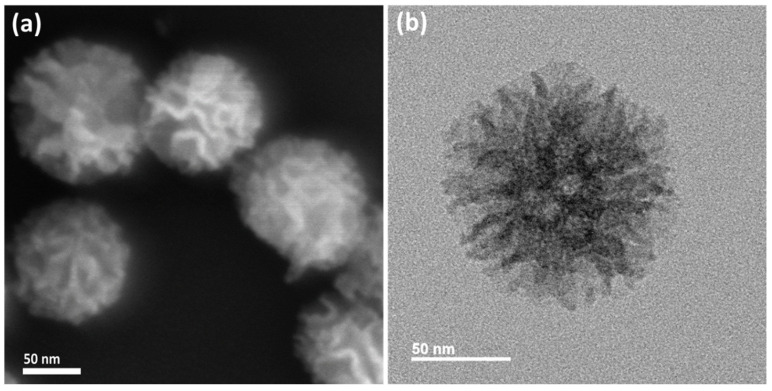
(**a**) FESEM and (**b**) FETEM images of LPMSN–amine.

The LPMSN–siloxane demonstrated a well-defined central-radial mesopore structure, showcasing uniform spherical morphology and large open pores, which were distinctly visible in high-magnification FESEM and FETEM images (refer to Figure S1a,b). In addition, LPMSN–amine also exhibited similar structures, as shown in Figure 1. This unique structural configuration resulted in significant BET surface areas and internal pore volumes due to the interconnected pores and highly convoluted walls. The LPMSNs displayed uniform small particles with central–radial mesopores, the size of which gradually increased from the inner to the outer regions. When observed under low magnification, the FETEM image revealed that LPMSN–amine was monodispersed, with an approximate mean diameter of 109 ± 10 nm and a polydispersity index of roughly 0.09 (as indicated in Figure S2).

**Figure 2:**
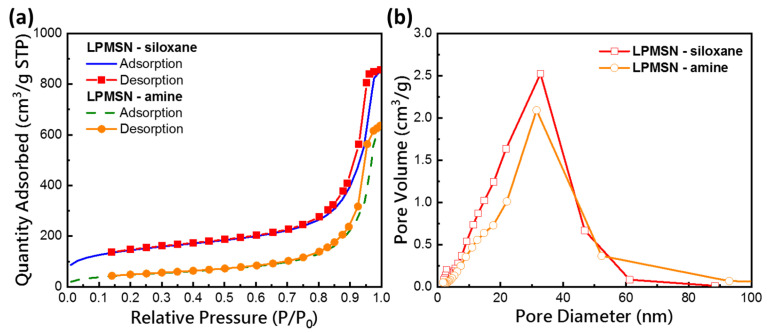
(**a**) N_2_ adsorption-desorption isotherm and (**b**) the corresponding pore size distribution curve of the LPMSN–siloxane and LPMSN–amine, respectively.

The average pore sizes and specific surface areas of the different functionalized LPMSNs were analyzed by using a high-resolution surface area analyzer and BET and BJH techniques. The typical type IV isotherm curve shown in Figure 2a indicated the presence of a mesoporous structure in the LPMSNs. The observed hysteresis loop in the adsorption–desorption curve of LPMSNs, classified as type H_1_, is commonly associated with materials possessing regular cylindrical or spherical pores or pores between regularly shaped particles, aiding in pore identification. The BET surface area (S_BET_), BET pore volume (V_P_), and BJH pore diameter (V_BJH_) of LPMSN–siloxane and LPMSN–amine are summarized in Table S1. Notably, surface area decreased from 518.21 m^2^/g to 176.80 m^2^/g following surface functionalization. However, a slight reduction in V_BJH_ to 31.6 nm (Figure 2b) compensated for the reduction in surface area [17]. The detailed characterization of the surface area and pore size of the functionalized LPMSNs provides valuable insights into their structural properties and supports their application as carriers for controlled HA delivery in the development of hydrogel CLs.

**Figure 3:**
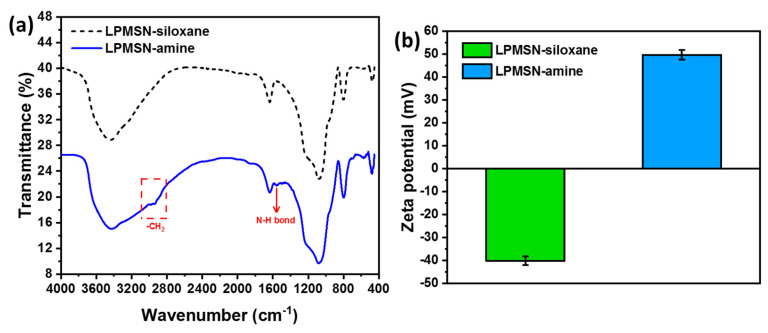
(**a**) FTIR spectra and (**b**) the corresponding zeta potential of LPMSN-siloxane and LPMSN–amine, respectively.

The FTIR spectra of LPMSNs with different functionalized groups are presented in Figure 3a. The spectra exhibited strong absorption signals at 1089 and 960 cm^−1^ that corresponded to the stretching and asymmetric stretching of Si–O–Si bridges and the skeletal vibration of C–O bonds, respectively. After modification with amines, a characteristic peak at 1561 cm^−1^ (associated with the stretching vibration of amide I and –NH_2_ bending) appeared, reflecting the coupling of NH_2_ groups to the surface of LPMSNs [17]. Additionally, the LPMSN–amine samples displayed a new absorption peak at 2800–2935 cm^−1^ that corresponded to the antisymmetric stretching vibration of –CH_2_ in the salinized APTES reagent. LPMSN–siloxane exhibited an absorption peak at 3300–3600 cm^−1^ attributable to the combined stretching vibrations of –OH and N–H. These spectral changes confirmed the successful grafting of the amine group onto the LPMSNs, thus verifying the formation of LPMSN–NH_2_. Furthermore, as shown in Figure 3b, the zeta potential of LPMSN–siloxane was measured to be −40.1 mV. However, upon the introduction of positively charged amine groups (LPMSN–amine, −NH_2_), the zeta potential significantly increased to 49.6 mV. This change in zeta potential provided strong evidence of successful modification with NH_2_ groups. Characterization through FTIR and zeta potential measurements validated the effective functionalization of LPMSNs with amine groups, a crucial step in enhancing drug-loading capacity and controlled release behavior. The positively charged surface of LPMSN–amine ensured strong interactions with HA molecules, facilitating efficient loading and sustained release, making the functionalized LPMSNs ideal candidates for HA delivery applications in our hydrogel CLs.

### 3.2. Characterization of LPMSN-Laden CLs

**Figure 4:**
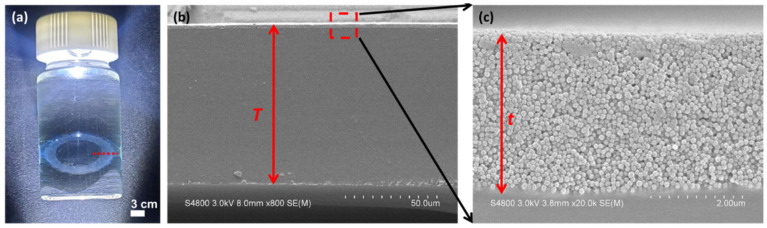
(**a**) Photograph of LPMSN–amine CLs soaking in the HA/PBS solution. (**b**) Cross-section FESEM images of the fabricated LPMSN–amine CLs, and (**c**) a higher magnification of the image in (**b**).

The LPMSN-laden CLs were crafted using cast molding with a PP lens mold, following procedures detailed in a previous study [20]. We introduced a circular HA delivery zone to retain the optical and physical attributes of the CLs, ensuring clarity in the optical zone. These resulting LPMSN-laden CLs had a 14 mm diameter (with a base curve of 8.5 mm) and featured a ring encircling the iris area, encompassing a transparent central optic zone of approximately 8.0 mm (as shown in Figure 4a). Remarkably, the inclusion of LPMSNs had minimal impact on the high optical transparency of all CLs, with all CLs maintaining over 90% transparency (see Figure S3). Additionally, they all met the requirements of class II UV blocking, effectively shielding against 70% of UV-A and 95% of UV-B radiation, as per the ANSI Z80.20 standard.

For a more comprehensive examination, please refer to Figure 4b,c for FESEM cross-section images displaying the LPMSN–amine ring embedded within the hydrogel CLs. Additionally, the LPMSN–amine was securely linked and anchored within the hydrogel matrix. These LPMSN–amine CLs showcased an initial center thickness of roughly 88 μm (*T*) before undergoing swelling through hydration. Simultaneously, the LPMSN–amine displayed an approximate thickness of 4 μm (*t*). The calculated volume ratio of LPMSN–amine within the dry CLs was approximately 1.6 vol%. It is essential to note that the physical, optical, and mechanical properties of all CLs in this study were in close alignment with those reported in our previous research [20]. The assessment of these properties in all CLs was performed in accordance with the Soft (Hydrophilic) Daily Wear Contact Lenses—Performance Criteria for Safety and Performance Based Pathway (FDA-2019-D-4843). These characterization results affirm that LPMSNs were effectively integrated into hydrogel CLs, preserving their optical clarity, UV-blocking capabilities, and structural integrity.

### 3.3. HA Loaded and Released of LPMSN-Laden CLs

We conducted HA loading and release experiments with standard, LPMSN–siloxane, and LPMSN–amine CLs. Each type of CL was immersed in 0.1 wt% HA/PBS solution (3 mL) with its ionic strength controlled at pH 5.5 and 6.5 for 24 h. The increased HA loading capacity observed in LPMSN-laden CLs in comparison to standard CLs suggests the effective loading of HA into the central-radial mesopores of LPMSNs. The mechanism of HA loading differed between LPMSN–siloxane and LPMSN–amine. LPMSN–siloxane achieved HA loading through hydrogen bonding, facilitated by the presence of silanol and siloxane groups within the mesopores. In contrast, LPMSN–amine loaded HA through a combination of hydrogen bonding between amine and hydroxyl groups, as well as charge adsorption (refer to Figure 3b). This dual interaction mechanism resulted in a higher HA loading capacity for LPMSN–amine compared to LPMSN–siloxane, as illustrated in Table S2. The pH within the mesopores of LPMSN–siloxane has previously been documented to be lower than that of the surrounding bulk solution [28]. It is possible that LPMSN–amine tends to attract more protons in a lower pH environment such as the HA/PBS solution. Consequently, LPMSN–amine-loaded CLs demonstrated a notably greater HA loading capacity at pH 5.5 as compared to pH 6.5.

**Figure 5:**
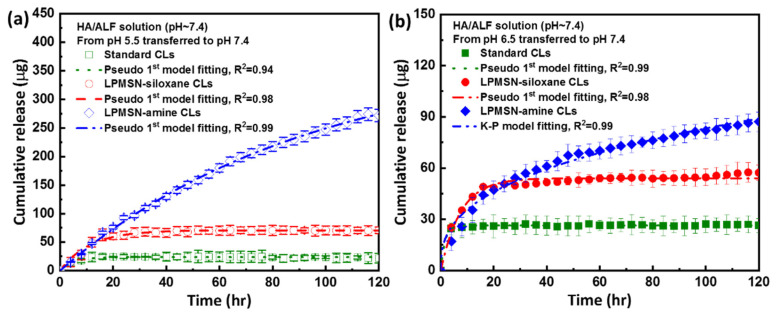
The cumulative release curves for (**a**) pH 5.5 and (**b**) pH 6.5 transferred to ALF solution are depicted for all samples. Please note that the time intervals on the X-scale correspond to the duration required to reach a stable release state. (*N* = 6).

After loading, the samples were transferred to 3 mL of ALF solution at pH 7.4 to facilitate the release of HA. We determined the HA concentration of ALF by using a UV–vis spectrophotometer to analyze the in vitro HA release profiles of the LPMSN-laden and standard CLs. The HA concentration of the ALF solution was measured over time as depicted in Figure 5. The absorbance values of the accumulated released samples over 120 h were converted into concentrations and fitted to a linear equation to determine the amount of HA released. Through these experiments, we aim to assess and compare the behavior of HA release from different CL types, elucidating the sustained release capabilities of the LPMSN-functionalized CLs. This information is critical for evaluating the potential of LPMSN-based hydrogel CLs as an advanced platform for the controlled ophthalmic delivery of HA and management of conditions, such as dry eye syndrome.

The total cumulative release (μg) of LPMSN-laden CLs prepared by using the soaking method is presented in Figure 5 and Table S2. Over a 120 h release period, the cumulative concentration of HA released from standard CLs in the ALF with pH adjusted to 7.4 was 21.9 μg at pH 5.5 and 26.4 μg at pH 6.5. By contrast, LPMSN–amine CLs demonstrated extended-release behavior with the maximum cumulative drug concentrations of 275.6 μg at pH 5.5 and 87.2 μg over 120 h at pH 6.5. For LPMSN–siloxane CLs, the corresponding maximum cumulative drug concentrations were 70.8 and 57.3 μg at pH 5.5 and 6.5, respectively. Notably, standard CLs exhibited very low HA release, whereas LPMSNs demonstrated significantly higher HA release than standard CLs. In particular, LPMSN–amine CLs showed a 12.6-fold (and 3.2-fold) increase in the amount of HA released compared with standard CLs (and LPMSN–siloxane CLs). In the case of HA release from standard CLs, there was a weakening of the interaction between the HA and poly(HEMA), leading to the desorption of the HA from the hydrogel sites. This phenomenon resulted in an increased degree of ionization [29,30], subsequently enhancing the repulsion between the gel chains. In contrast, the ionization of the silanol, siloxane, and amine groups within LPMSN-laden CLs was influenced by the pH change when the LPMSN-laden CLs were shifted from the soaking solution (pH 5.5 or 6.5) to ALF environment. This change in pH conditions reduced the binding affinity of HA and facilitated the gradual release of HA from the LPMSN-laden CLs. Additionally, the amine groups of LPMSN–amine may undergo protonation, leading to the transformation of the hydrophobic mesopore interior into a hydrophilic one, thereby further facilitating the release of internal HA. In addition, the high released amount of HA in LPMSN–amine CLs can be attributed to the strong electrostatic attraction between the positively charged LPMSN–amine and the negatively charged HA, as depicted in Figure 3b. However, the presence of HA might hinder penetration into the pore channels of LPMSN–siloxane because both carry negative charges of HA and LPMSN–siloxane. These results highlight the exceptional drug-loading and sustained-release capabilities of LPMSN–amine CLs, confirming their potential as advanced carriers for controlled ophthalmic drug delivery. The significant increase in HA release from LPMSN–amine CLs relative to that from standard CLs and LPMSN–siloxane CLs offers promising prospects for enhancing the treatment of dry eye syndrome and other ocular conditions through improved drug delivery efficiency and prolonged therapeutic effects.

Additionally, the substantial amount of rapidly released HA from standard CLs during the initial 4 h (burst release) indicated that adsorbed HA was released from the surface of the hydrogel sheets. This phenomenon suggested that standard CLs do not effectively sustain HA release likely due to the hindrance caused by the high molecular weight of HA, which limited the penetration of HA into the aqueous channels of the hydrogel sheets. As a result, HA remained adsorbed only on the surface, leading to its rapid release into the surrounding media. These findings shed light on the limitations of the soaking method for ocular drug delivery via LPMSN-laden CLs, particularly when addressing high-molecular-weight polymers, such as HA. This situation indicated the potential benefits of employing implantable hydrogel CLs for extended wear in the treatment of KCS because such CLs can offer enhanced control over drug release and minimize the burst release effect. Sustained and controlled drug delivery can be achieved by using implantable hydrogel CLs, addressing the limitations observed with the soaking method. Moreover, the use of LPMSNs in the hydrogel CLs offers an innovative approach for enhancing therapeutic outcomes in the management of dry eye syndrome. These advances hold promise for overcoming the challenges associated with traditional drug delivery methods, making LPMSN-laden hydrogel CLs a valuable candidate for improving patient comfort and treatment efficacy.

### 3.4. HA Released Kinetics Mechanism of LPMSN-Laden CLs

The release measurement results in Figure 5 demonstrate that the standard CLs exhibited the burst release of HA, likely due to the adsorption of HA on their outer surface. Upon contact with the release environment at pH 7.4, the adsorbed HA did not require diffusion for release; instead, it directly dissolved in the release medium, leading to quick release. By contrast, LPMSNs demonstrated slow-release behavior. HA release from LPMSNs involved the penetration of the release medium into the mesopores of LPMSNs, followed by the dissolution of HA in the release medium. The concentration gradient drove the osmotic pressure, enabling HA to diffuse out of the mesopores of LPMSNs. Additionally, the pore size of LPMSNs and the affinity between HA and LPMSNs significantly influenced HA release. As a result of the strong affinity between HA and LPMSN–amine DCLs, the HA release rate was slow. We performed kinetic modeling analysis, and the results are presented in Tables S3 and S4. For standard CLs, LPMSN–siloxane CLs, and LPMSN–amine CLs of the release of HA from pH 5.5 transferred to ALF followed the pseudo-first-order model and its mechanism was diffusion-controlled. By contrast, pH 6.5 transferred to ALF of HA release from LPMSN–amine CLs followed the K–P model as shown in Table S4. The analysis yielded the following results: The release mechanism after loading the drug at pH 6.5 and transferring it to pH 7.4 for drug release was Fickian diffusion-controlled release (*n* < 0.45). The HA release mechanism of LPMSN–amine is primarily diffusion-based, since the control of release is mainly governed by the amine groups within the mesopores, undergoing hydrophilic–hydrophobic transitions under different pH environments. The unique structure and properties of LPMSN–amine play a crucial role in sustained drug release, with the amine functional groups providing favorable interactions with HA molecules. These findings provide valuable insights into the release kinetics of HA from LPMSN-laden CLs, highlighting the importance of understanding the interactions between drug-loaded nanoparticles and the hydrogel matrix. The observed slow-release behavior of LPMSN–amine CLs offers the potential for prolonged and controlled drug delivery, making them promising candidates for therapeutic applications, especially in the treatment of dry eye syndrome.

### 3.5. Wetting Angle of LPMSN-Laden CLs with HA

**Figure 6:**
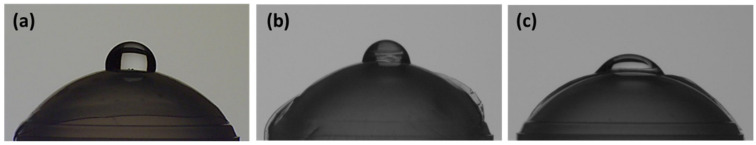
Water contact angle photographs of (**a**) the standard, (**b**) the LPMSN–siloxane, and (**c**) the LPMSN–amine CLs after HA soaking, respectively.

HA, when present in the hydrogel sheet, contributes to increased hydration, ion and oxygen permeability, and overall comfort during wear because of its hydrophilic nature. Water contact angle measurements were performed to assess the hydrophilicity of the CL surface after standard CLs were modified with LPMSNs. Unloaded standard CLs exhibited a water contact angle of approximately 90.5°, whereas LPMSN–amine (and LPMSN–siloxane) showed a reduced angle of 65.8° (and 80.1°), as displayed in Figure S4. However, after soaking in HA, the water contact angle of standard CLs decreased to approximately 90.0°, whereas that of LPMSN–amine (and LPMSN–siloxane) further reduced to 45.3° (and 75.0°), as shown in Figure 6. These results demonstrated that the introduction of LPMSNs and HA increased the hydrophilicity and wettability of standard CLs. This enhancement in hydrophilicity suggested that modification can improve the ability to entrap HA molecules and enhance the water absorbency of CLs. The increased hydrophilicity of LPMSN-laden CLs is likely to enhance water retention and reduce dehydration at the ocular surface. This effect, in turn, can contribute to improved patient comfort during prolonged wear and may be particularly beneficial in managing dry eye syndrome, wherein maintaining adequate moisture on the ocular surface is crucial for alleviating symptoms. Overall, the combination of LPMSNs and HA in the hydrogel matrix holds promise for the development of advanced CLs with enhanced hydrophilicity and prolonged drug release capabilities, providing potential benefits for ocular health and patient well-being.

## 4. Conclusions

We successfully developed extended-release hydrogel CLs embedded with LPMSNs for the sustained delivery of HA. LPMSN–amine CLs exhibited significantly improved HA loading capacity and controlled release compared with LPMSN–siloxane and standard CLs. The use of LPMSN–amine CLs allowed for the increased loading and sustained release of HA owing to the strong electrostatic attraction between the positively charged LPMSN–amine and the negatively charged HA. HA releasing efficiency of LPMSN–amine notably increased by 12.6-fold at pH 5.5 compared to standard CLs. Furthermore, we demonstrated the promising potential of extended-release LPMSN-embedded hydrogel CLs for HA delivery without compromising the optical and physical properties of CLs. Our findings demonstrated the potential of LPMSN–amine CLs as a promising platform for managing dry eye syndrome and other ocular conditions requiring prolonged drug release. These findings are promising and provide new possibilities for developing advanced CLs that are capable of delivering HA in a sustained manner, potentially revolutionizing the treatment of conditions such as KCS and considerably enhancing patient comfort and therapeutic outcomes. Such a development holds great potential for enhancing the treatment of KCS and providing improved comfort and relief to patients.

## Data Availability

Data supporting reported results can be found in the Supplementary Materials.

## Supplementary Materials

The following supporting information can be downloaded at: https://www.mdpi.com/article/10.3390/nano13172441/s1, Table S1. The N_2_ adsorption–desorption parameters of different functionalized LPMSNs.; Table S2. HA/PBS solution with different pH values for all CLs.; Table S3. Kinetic fitting data of desorption between hydrogels and HA for pH 5.5.; Table S4. Kinetic fitting data of desorption between hydrogels and HA for pH 6.5.; Figure S1. (a) FESEM and (b) FETEM images of the LPMSN–siloxane.; Figure S2. Particle size distribution calculated from FETEM pictures. (a) FETEM image and (b) histograms of particle size distribution of LPMSN–amine.; Figure S3. Transmittance spectra of the fabricated CLs. Inset shows the transmittance of different wavelength ranges. (*N* = 6).; Figure S4. Water contact angle photographs of (a) the standard, (b) the LPMSN–siloxane, and (c) the LPMSN–amine CLs without HA, respectively.

## References

[1] Farandos N.M., Yetisen A.K., Monteiro M.J., Lowe C.R., Yun S.H. (2015). Contact lens sensors in ocular diagnostics. Adv. Healthc. Mater..

[2] Ali M., Horikawa S., Venkatesh S., Saha J., Hong J.W., Byrne M.E. (2007). Zero-order therapeutic release from imprinted hydrogel contact lenses within in vitro physiological ocular tear flow. J. Control. Release.

[3] Cooper R.C., Yang H. (2019). Hydrogel-based ocular drug delivery systems: Emerging fabrication strategies, applications, and bench-to-bedside manufacturing considerations. J. Control. Release.

[4] Torres-Luna C., Hu N., Tammareddy T., Domszy R., Yang J., Wang N.S., Yang A. (2019). Extended delivery of non-steroidal anti-inflammatory drugs through contact lenses loaded with Vitamin E and cationic surfactants. Contact Lens Anterior Eye.

[5] Paradiso P., Serro A.P., Saramago B., Colaço R., Chauhan A. (2016). Controlled release of antibiotics from vitamin E–loaded silicone-hydrogel contact lenses. J. Pharm. Sci..

[6] Hong S., Yang K., Kang B., Lee C., Song I.T., Byun E., Park K.I., Cho S.W., Lee H. (2013). Hyaluronic acid catechol: A biopolymer exhibiting a pH-dependent adhesive or cohesive property for human neural stem cell engineering. Adv. Funct. Mater..

[7] Bukhari S.N.A., Roswandi N.L., Waqas M., Habib H., Hussain F., Khan S., Sohail M., Ramli N.A., Thu H.E., Hussain Z. (2018). Hyaluronic acid, a promising skin rejuvenating biomedicine: A review of recent updates and pre-clinical and clinical investigations on cosmetic and nutricosmetic effects. Int. J. Biol. Macromol..

[8] Gulsen D., Chauhan A. (2004). Ophthalmic drug delivery through contact lenses. Investig. Ophthalmol. Vis. Sci..

[9] Hsu K.H., Carbia B.E., Plummer C., Chauhan A. (2015). Dual drug delivery from vitamin E loaded contact lenses for glaucoma therapy. Eur. J. Pharm. Biopharm..

[10] Maulvi F.A., Soni T.G., Shah D.O. (2016). A review on therapeutic contact lenses for ocular drug delivery. Drug Deliv..

[11] Maulvi F.A., Mangukiya M.A., Patel P.A., Vaidya R.J., Koli A.R., Ranch K.M. (2016). Extended release of ketotifen from silica shell nanoparticle-laden hydrogel contact lenses: In vitro and in vivo evaluation. J. Mater. Sci. Mater. Med..

[12] Siripongpreda T., Pongsachareonnont P., Chanajaree R., Rodthongkum N. (2023). Theranostic contact lens based on cellulose nanofibrils/levofloxacin nanocomposite for ocular bacterial infection. Cellulose.

[13] Dong H., Jin X.T., Zhang S.X., Xue C., Liu M., Luo Y.H. (2023). Colored contact lens with all-weather humidity-triggered discoloration. Dye. Pigment..

[14] Hisham M., Salih A.E., Butt H. (2023). 3D Printing of Multimaterial Contact Lenses. ACS Biomater. Sci. Eng..

[15] Jooken S., Deschaume O., Bartic C. (2023). Nanocomposite Hydrogels as Functional Extracellular Matrices. Gels.

[16] Abu-Dief A.M., Alsehli M., AI-Enizi A., Nafady A. (2022). Recent advances in mesoporous silica nanoparticles for targeted drug delivery applications. Curr. Drug Deliv..

[17] Zhang J., Sun Y., Tian B., Li K., Wang L., Liang Y., Han J. (2016). Multifunctional mesoporous silica nanoparticles modified with tumor-shedable hyaluronic acid as carriers for doxorubicin. Colloids Surf. B Biointerfaces.

[18] Li M., Liang Y., He J., Zhang H., Guo B. (2020). Two-pronged strategy of biomechanically active and biochemically multifunctional hydrogel wound dressing to accelerate wound closure and wound healing. Chem. Mater..

[19] Manzano M., Vallet-Regi M. (2020). Mesoporous silica nanoparticles for drug delivery. Adv. Funct. Mater..

[20] Lai C.F., Shiau F.J. (2023). Enhanced and Extended Ophthalmic Drug Delivery by pH-Triggered Drug-Eluting Contact Lenses with Large-Pore Mesoporous Silica Nanoparticles. ACS Appl. Mater. Interfaces.

[21] Rong H., Dong Y., Zhao J., Zhang X., Li S., Sun Y., Lu T., Yu S., Hu H. (2023). Fetal milieu-simulating hyaluronic acid-dopamine-chondroitin sulfate hydrogel promoting angiogenesis and hair regeneration for wound healing. Int. J. Biol. Macromol..

[22] Liu W., Liu Y.Y., Zhang M.Q., Qin M.Z., Yang Y.Y., Liu B.W., Zhang D.J., Jiang C.H., Yin Z.Q., Lu M. (2023). A comparative study of the ameliorative effects of hyaluronic acid oligosaccharides and hyaluronic acid on DSS-induced colitis in mice and research on relevant mechanisms. Food Funct..

[23] Lai C.F., Li J.S., Fang Y.T., Chien C.J., Lee C.H. (2018). UV and Blue−Light Anti−Reflective Structurally Colored Contact Lenses based on a Copolymer Hydrogel with Amorphous Array Nanostructures. RSC Adv..

[24] Deng J., Chen S., Chen J., Ding H., Deng D., Xie Z. (2018). Self-reporting colorimetric analysis of drug release by molecular imprinted structural color contact lens. ACS Appl. Mater. Interfaces.

[25] Grillo R., Pereira A.d.E.S., Melo N.F.S.d., Porto R.M., Feitosa L.O., Tonello P.S., Filho N.L.D., Rosa A.H., Lima R., Fraceto L.F. (2011). Controlled release system for ametryn using polymer microspheres: Preparation, characterization and release kinetics in water. J. Hazard. Mater..

[26] Tieppo A., Boggs A.C., Pourjavad P., Byrne M.E. (2014). Analysis of release kinetics of ocular therapeutics from drug releasing contact lenses: Best methods and practices to advance the field. Contact Lens Anterior Eye.

[27] Chu Z., Xue C., Shao K., Xiang L., Zhao X., Chen C., Pan J., Lin D. (2022). Photonic Crystal−Embedded Molecularly Imprinted Contact Lenses for Controlled Drug Release. ACS Appl. Bio Mater..

[28] Zhu Y., Derami H.G., Gupta P., Singamaneni S., Jun Y.S. (2022). Ionic surface propensity controls pH in nanopores. Chem.

[29] Kovaleva E.G., Molochnikov L.S., Venkatesan U., Marek A., Stepanova D.P., Kozhikhova K.V., Mironov M.A., Smirnov A.I. (2016). Acid–base properties of nanoconfined volumes of anodic aluminum oxide pores by EPR of pH-sensitive spin probes. J. Phys. Chem. C.

[30] Wang F., Widejko R.G., Yang Z., Nguyen K.T., Chen H., Fernando L.P., Christensen K.A., Anker J.N. (2012). Surface-enhanced Raman scattering detection of pH with silica-encapsulated 4-mercaptobenzoic acid-functionalized silver nanoparticles. Anal. Chem..

