# Supplementary material for: Hydrogel Contact Lenses Embedded with Amine-Functionalized Large-Pore Mesoporous Silica Nanoparticles with Extended Hyaluronic Acid Release
Source: Nanomaterials (Basel). 2023 Aug 28;13(17):2441. doi: 10.3390/nano13172441 (PMC10490223; doi:10.3390/nano13172441)
Supplement: Supplementary file 1 [file nanomaterials-13-02441-s001.zip › nanomaterials-2577838-supplementary.pdf]

*Supporting Information*

# Hydrogel Contact Lenses Embedded with Amine-Functionalized Large-Pore Mesoporous Silica Nanoparticles with Extended Hyaluronic Acid Release

**Chun-Feng Lai * and Fu-Jia Shiau**

Department of Photonics, Feng Chia University, No. 100, Wenhwa Road, Seatwen, Taichung 40724, Taiwan.
* Correspondence: author: chunflai@fcu.edu.tw

**Table S1.** The $N_2$ adsorption–desorption parameters of different functionalized LPMSNs.

| Sample | BET surface area $S_{BET}$ ($m^2/g$) | BET pore volume Vp ($cm^3/g$) | BJH pore diameter $V_{BJH}$ (nm) |
|---|---|---|---|
| LPMSN-siloxane | 518.21 | 1.20 | 33.1 |
| LPMSNs-amine | 176.80 | 0.76 | 31.6 |

**Abbreviations:** BET, Brunauer-Emmett-Teller; BJH, Barrett-Joyner-Halenda.

**Table S2.** HA/PBS solution with different pH values for all CLs.

| Sample | Uptake pH Environment | Loaded HA on 24 h [μg/lens][a)] | Total Released HA in ALF on 120 h [μg/lens] | Released 80% HA Drug | Released HA Enhancement[b)] |
|---|---|---|---|---|---|
| Standard CLs | 5.5 | 25.7 | 21.9 | 8 h | — |
| | 6.5 | 30.2 | 26.4 | 3 h | — |
| LPMSN-siloxane CLs | 5.5 | 80.3 | 70.8 | 13 h | 3.2-fold |
| | 6.5 | 65.5 | 57.3 | 10 h | 2.2-fold |
| LPMSN-amine CLs | 5.5 | 281.2 | 275.6 | 82 h | 12.6-fold |
| | 6.5 | 95.6 | 87.2 | 60 h | 3.3-fold |

[a)] HA/PBS solution with different pH value is fixed at 0.1 wt% carried out 3 mL for uptake. [b)] Released HA enhancement values are calculated by comparing them to standard CLs.

**Table S3.** Kinetic fitting data of desorption between hydrogels and HA for pH 5.5.

| Sample | Pseudo First-Order Model | | | Korsmeyer-Peppas Model | | |
|---|---|---|---|---|---|---|
| | $k_1$ | $Q_e$ | $R^2$ | $n$ | $k_{kp}$ | $R^2$ |
| Standard CLs | 0.20±0.02 | 23.78±0.23 | 0.94 | 0.06±0.02 | 18.55±1.74 | 0.77 |
| LPMSN-siloxane CLs | 0.09±0.00 | 70.75±0.59 | 0.98 | 0.19±0.03 | 30.56±3.81 | 0.82 |
| LPMSN-amine CLs | 0.01±0.00 | 379.61±6.63 | 0.999 | 0.71±0.01 | 9.57±0.57 | 0.995 |

**Table S4.** Kinetic fitting data of desorption between hydrogels and HA for pH 6.5.

| Sample | Pseudo first-order model | | | Korsmeyer-Peppas model | | |
|---|---|---|---|---|---|---|
| | $k_1$ | $Q_e$ | $R^2$ | $n$ | $k_{kp}$ | $R^2$ |
| **Standard CLs** | **0.63±0.06** | **26.35±0.08** | **0.99** | **0.02±0.0** | **24.32±0.33** | **0.98** |
| **LPMSN-siloxane CLs** | **0.13±0.01** | **54.01±0.36** | **0.98** | **0.14±0.01** | **29.60±1.72** | **0.94** |
| **LPMSN-amine CLs** | **24.94±0.00** | **66.23±3.30** | **0.31** | **0.37±0.01** | **15.31±0.82** | **0.99** |

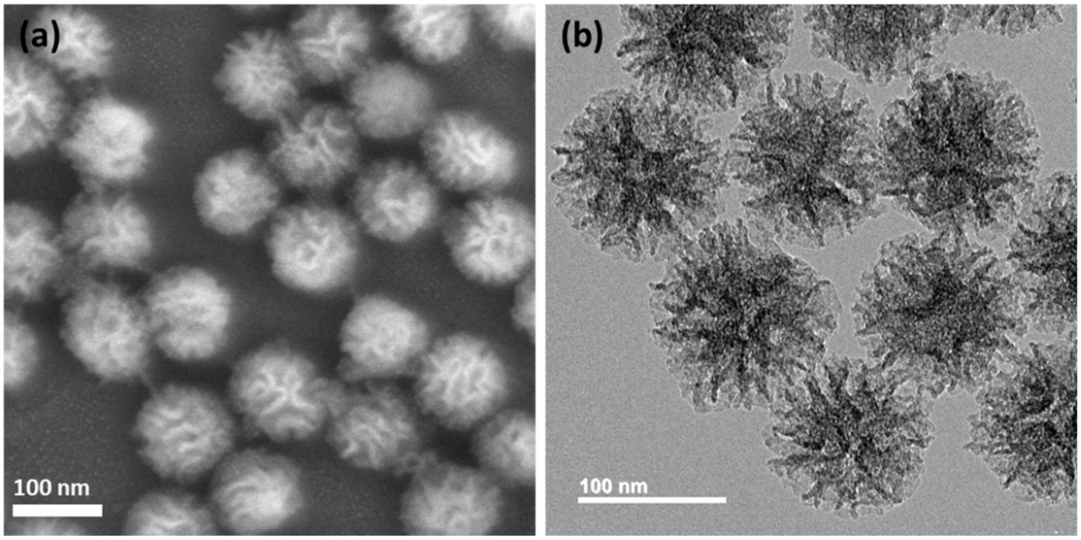


**Figure S1.** (a) FESEM and (b) FETEM images of the LPMSN-siloxane.

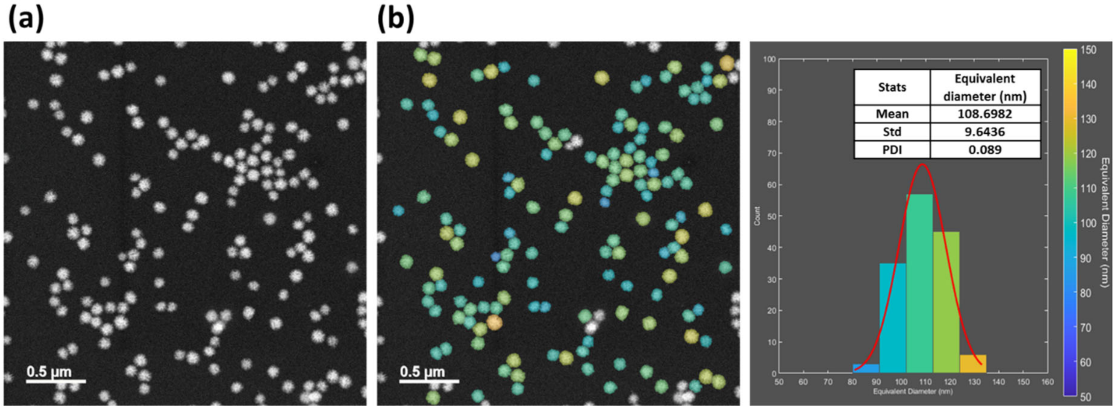


**Figure S2.** Particle size distribution calculated from FETEM pictures. (a) FETEM image and (b) histograms of particle size distribution of LPMSN-amine.

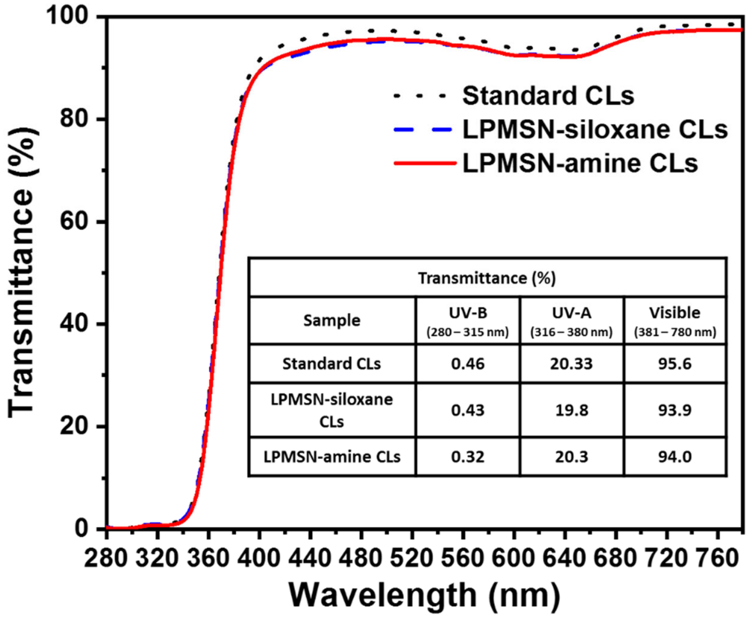


| Transmittance (%) | | | |
| --- | --- | --- | --- |
| Sample | UV-B (280 – 315 nm) | UV-A (316 – 380 nm) | Visible (381 – 780 nm) |
| Standard CLs | 0.46 | 20.33 | 95.6 |
| LPMSN-siloxane CLs | 0.43 | 19.8 | 93.9 |
| LPMSN-amine CLs | 0.32 | 20.3 | 94.0 |

**Figure S3.** Transmittance spectra of the all fabricated CLs. Inset shown the transmittance of different wavelength range. (Average, *N* = 6).

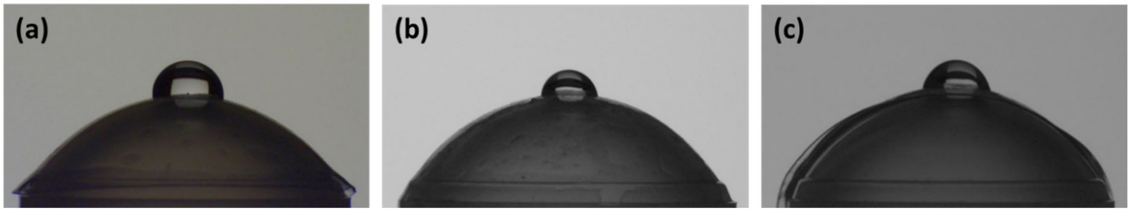


**Figure S4.** Water contact angle photographs of (a) the standard, (b) the LPMSN-siloxane, and (c) the LPMSN-amine CLs without HA, respectively.
